# Data on quality indices of groundwater resource for agricultural use in the Jolfa, East Azerbaijan, Iran

**DOI:** 10.1016/j.dib.2018.06.017

**Published:** 2018-06-19

**Authors:** Zoha Heidarinejad, Bayram Hashemzadeh, Ghasem Kiani Feizabadi, Farzaneh Baghal Asghari, Majid Radfard, Bahman Akbarpour, Hossein Najafi Saleh, Hossein Faraji

**Affiliations:** a Department of Environmental Health Engineering, Faculty of Health, Hormozgan University of Medical Sciences, Bandar Abbas, Iran; b Khoy University of Medical Sciences, Khoy, Iran; c Department of Environmental Health Engineering, School of Health, Semnan University of Medical Sciences, Semnan, Iran; d Department of Environmental Health Engineering, School of public Health, Tehran University of Medical Sciences, Tehran, Iran; e Health Research Center, Baqiyatallah University of Medical Sciences, Tehran, Iran; f Department of Environmental Health Engineering, Torbat Heydariyeh University of Medical Sciences, Torbat Heydariyeh, Iran; g Students Research Committee, Hamadan University of Medical Sciences, Hamadan, Iran

**Keywords:** Groundwater, Indices changes, SAR, Na%, Jolfa

## Abstract

The aim of this study was to evaluate the groundwater quality Indices of Groundwater resource for Agricultural Use in jolfa city (Iran) during one decade (2003–2013). Data showed in the first and end year of the study period, the Mean±SD of Sodium Adsorption Ratio (SAR) and Sodium Percentage (Na%) indices 5455.77±3878.02, 3638.69±3565.19 and 51.49±15.65, 41.58±17.69, respectively. The data indicate that the, in terms of sodium percentage and sodium adsorption ratio, the water quality in this area is not suitable for irrigation.

**Specifications Table**

**Table:** 

Subject area	Chemistry
More specific subject area	Chemistry of groundwater
Type of data	Table, Figures
How data was acquired	Data collected from Iran Water Resources Management Organization during the years 2003– 2013
Data format	Raw, analyzed
Experimental factors	All water samples were stored in a polyethylene bottles at room temperature in dark place.
Data source location	Jolfa, East Azerbaijan province, Iran
Data accessibility	The data are available with this article

**Value of the data**

• Determination of the physical and chemical quality of the groundwater resources of the city of Jolfa, East Azerbaijan province, Iran.
• Determine the indices changes in the quality of water resources in the city quality and management of water resources to prevent significant risks to human health [1], [2], [3], [4], [5], [6], [7], [8], [9].
• The result of data analysis shows that ground water in this some area is not suitable for agricultural according to calculated indices.
• The data of this study can help to better understand the quality of groundwater in the area and provide further studies.

## 1 Data

Data presented here deal with monitoring of physical and chemical including pH, Na^+^, Ca^2+^, Mg^2+^, K^+^, EC, TDS, ${HCO}_{3}^{-}$, ${SO}_{2}^{4-}$, Cl^−^, and TH as shown in Table 2. Results of water Na% and SAR indices calculations samples obtained from Jolfa city shown in Table 3. Summary of water quality indices in present study presented in Table 1.

**Table 1:** Summary of water quality indices in present study [10], [11], [12], [13], [14].

Indices	Formula
Sodium percentage (Na %)	$\mathrm{Na}\%=\frac{\mathrm{Na}+K}{\mathrm{Ca}+\mathrm{Mg}+\mathrm{Na}+K}\times \;100$
Sodium adsorption ratio (SAR)	$\mathrm{SAR}=\frac{\mathrm{Na}}{\sqrt{(\mathrm{Ca}+\mathrm{Mg})/2}}\times 100$

**Table 2:** Chemical analysis report of water quality of drinking water resource of Jolfa city.

Year	pH	Na^+^	Ca^2+^	Mg^2+^	K^+^	${HCO}_{3}^{-}$	${SO}_{4}^{2-}$	Cl^−^	TDS	EC	TH
		mg/L	mg/L	mg/L	mg/L	mg/L	mg/L	mg/L	mg/L	mg/L	mg/L as CaCO_3_
2003	7.65	279.78	89.21	64.88	6.70	359.86	315.77	349.44	733.25	1126.85	491.12
2004	7.51	251.46	112.54	79.19	6.94	472.36	246.67	369.61	754.67	1166.38	608.56
2005	7.45	256.88	109.40	69.47	6.18	428.84	218.68	388.51	715.25	1128.45	554.80
2006	7.50	260.85	105.63	70.76	6.42	421.63	230.63	387.46	679.75	1132.92	556.45
2007	7.76	241.64	101.90	67.52	5.87	384.53	226.32	363.52	640.77	1067.95	533.78
2008	7.97	240.87	96.13	74.69	5.90	388.32	230.52	365.23	648.78	1081.31	548.97
2009	7.66	218.20	91.55	63.11	5.59	325.27	215.00	340.96	583.66	972.76	489.65
2010	7.85	240.98	104.06	69.12	5.07	410.53	214.80	363.10	646.60	1077.67	545.75
2011	8.17	213.20	76.45	61.04	4.44	391.75	158.44	297.13	548.92	1023.81	443.35
2012	8.02	225.44	86.45	64.00	4.03	418.44	177.93	314.34	588.97	978.00	480.60
2013	7.55	201.48	89.11	67.41	4.19	455.27	165.32	281.20	567.43	945.71	501.31
Mean	7.74	241.44	97.87	68.55	5.61	405.33	220.80	351.86	651.77	1071.98	527.42
Max	9.10	825.50	417.00	191.18	12.62	947.12	873.60	843.00	1062.20	1770.00	905.40
Min	6.30	115.00	12.40	17.55	2.80	131.15	48.00	99.40	124.80	208.00	275.00
SD	0.49	234.49	90.60	38.76	3.70	174.42	203.18	387.84	461.71	762.93	339.81
WHO Guide Line	6.5–8.5	400	250	150	–	–	200	200	500.00	–	200.00
1053IR Standard	6.5–8.6	200	300	30	–	–	250	250	1000.00	–	200.00

**Table 3:** Results of water Na% and SAR indices calculations samples obtained from Jolfa city.

Year	SAR	Na%
2003	5455.77	51.49
2004	4273.21	41.38
2005	4532.03	43.96
2006	4615.19	45.21
2007	4330.70	44.07
2008	4248.65	43.22
2009	4019.50	42.18
2010	4237.66	42.56
2011	3887.75	40.20
2012	4028.86	41.60
2013	3638.69	41.58
Mean	4328.59	43.48

Na% and SAR indices in groundwater resources, also physical and chemical parameters in the city of Jolfa in the years (2003–2013) shown in Fig. 2, Fig. 3, Fig. 4, Fig. 5, Fig. 6, Fig. 7, Fig. 8, Fig. 9, Fig. 10, Fig. 11, Fig. 12, Fig. 13.

**Fig. 1:**
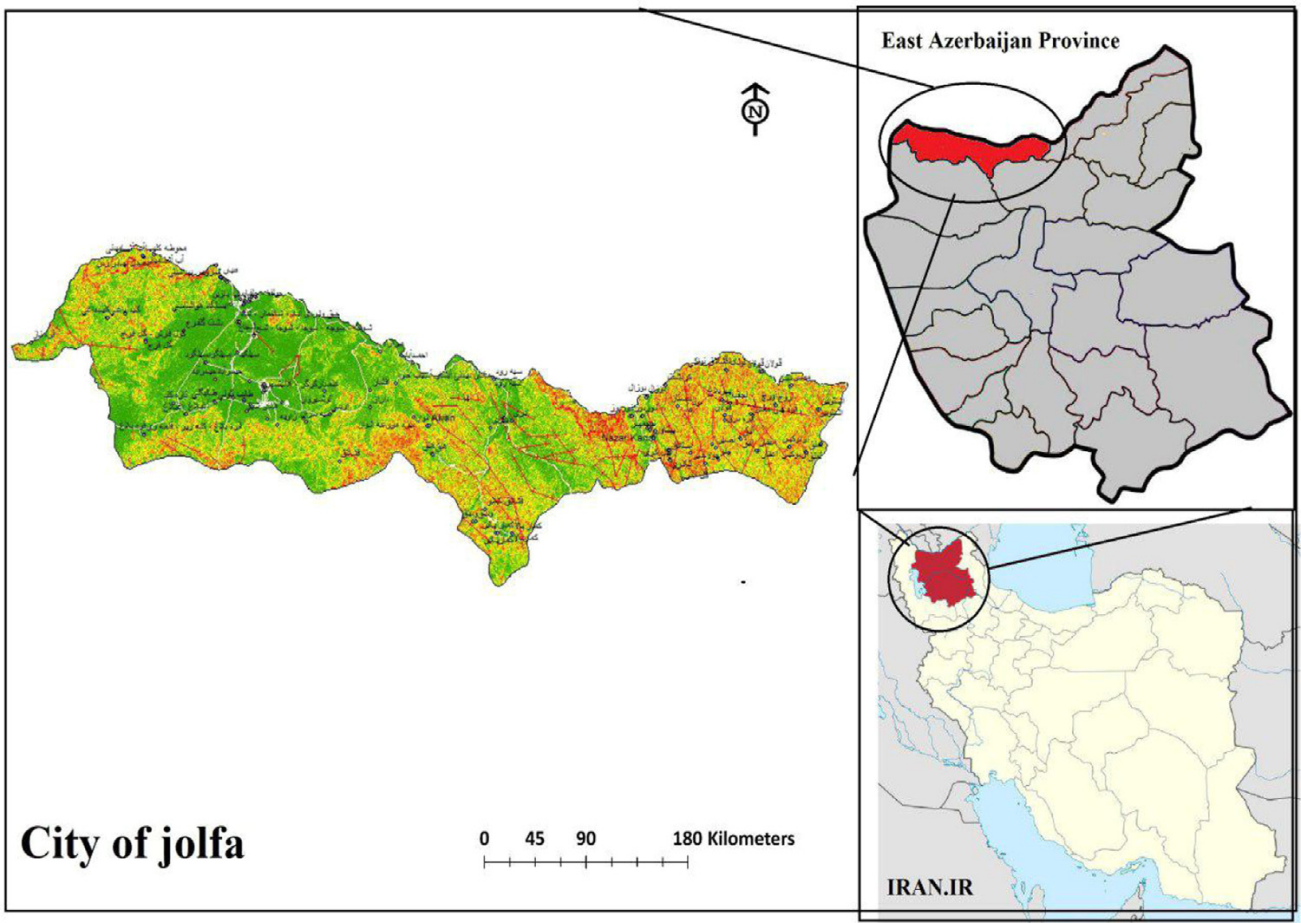
Location of the study area in Jolfa city, East Azerbaijan, Iran.

**Fig. 2:**
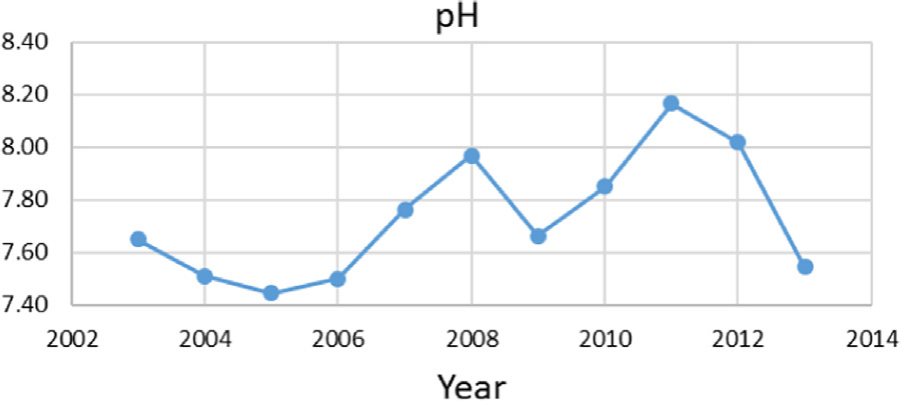
pH parameter for ground water in Jolfa city (During 2003–2013).

**Fig. 3:**
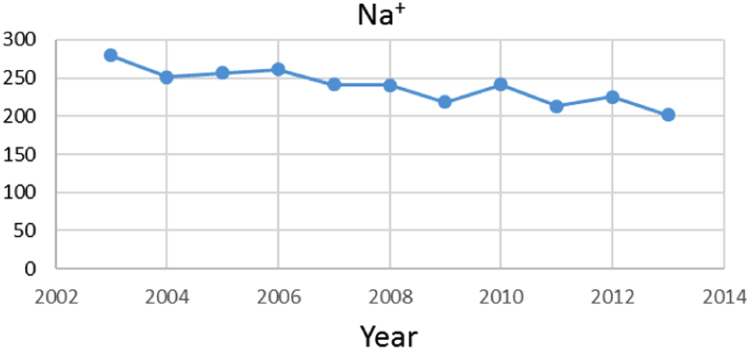
Na^+^ parameter for ground water in Jolfa city (During 2003–2013).

**Fig. 4:**
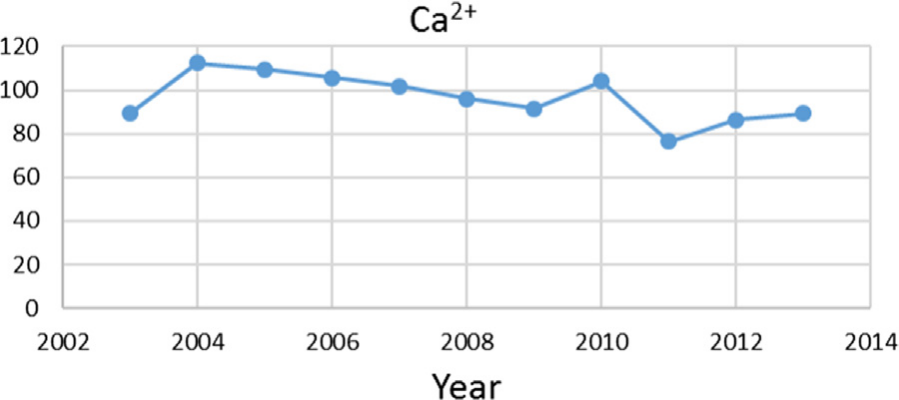
Ca^2+^ parameter for ground water in Jolfa city (During 2003–2013).

**Fig. 5:**
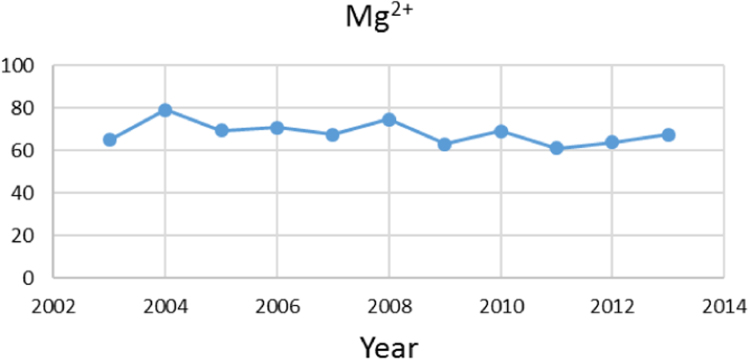
Mg^2+^ parameter for ground water in Jolfa city (During 2003–2013).

**Fig. 6:**
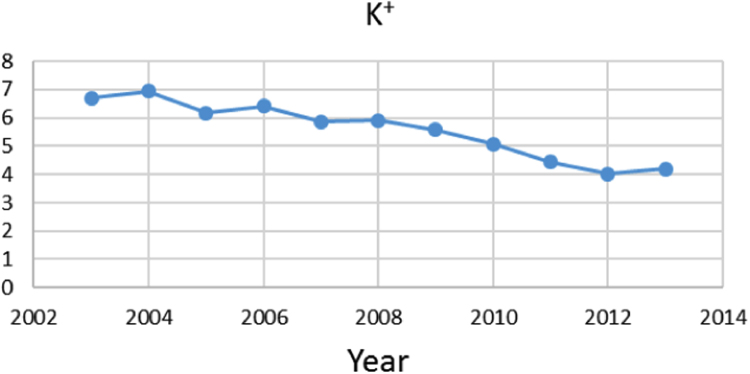
K^+^ parameter for ground water in Jolfa city (During 2003–2013).

**Fig. 7:**
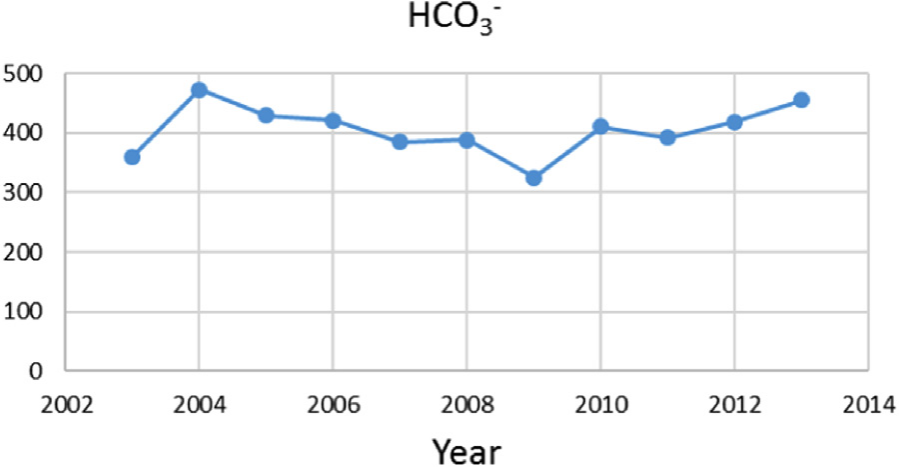
${HCO}_{3}^{-}$ parameter for ground water in Jolfa city (During 2003–2013).

**Fig. 8:**
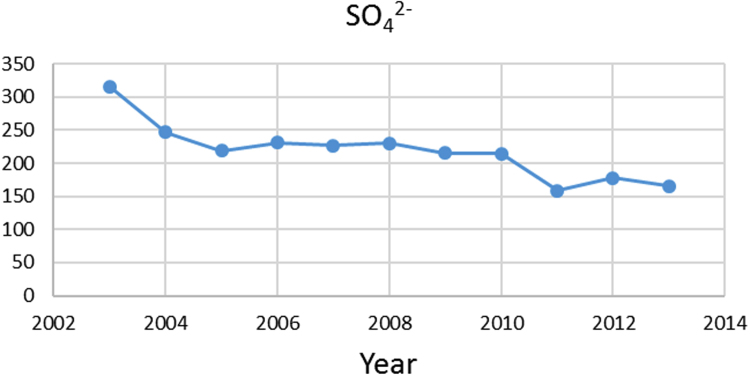
${SO}_{4}^{2-}$ parameter for ground water in Jolfa city (During 2003–2013).

**Fig. 9:**
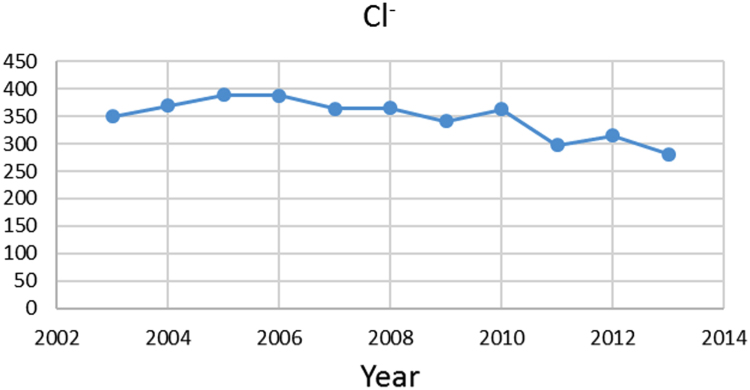
Cl^−^ parameter for ground water in Jolfa city (During 2003–2013).

**Fig. 10:**
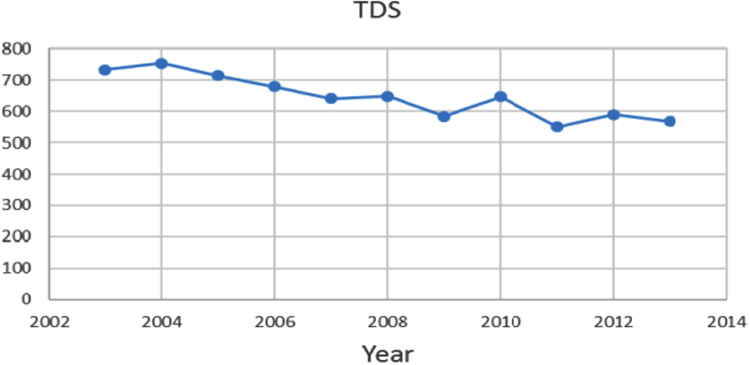
TDS parameter for ground water in Jolfa city (During 2003–2013).

**Fig. 11:**
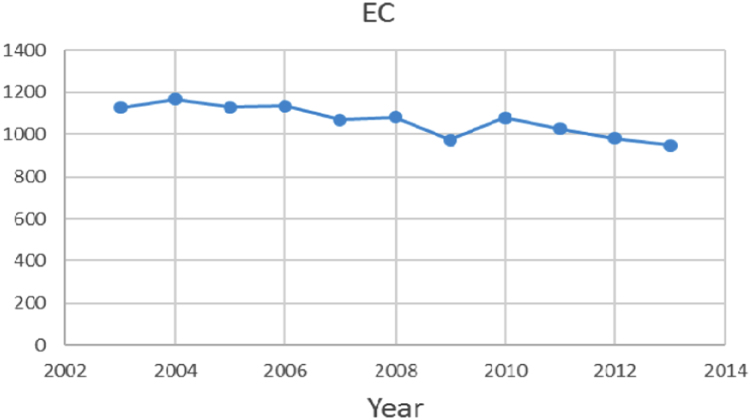
EC parameter for ground water in Jolfa city (During 2003–2013).

**Fig. 12:**
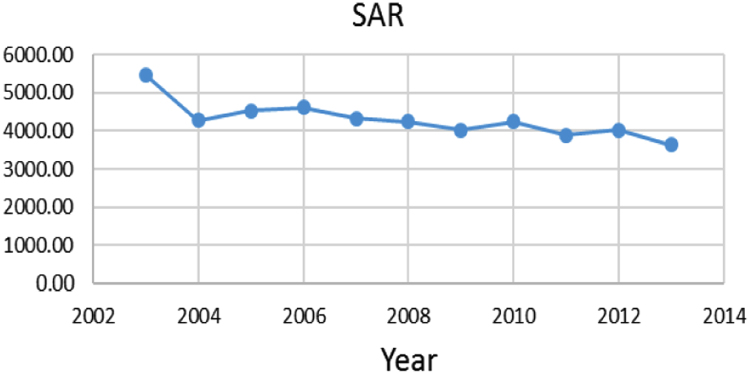
SAR index for ground water in Jolfa city (During 2003–2013).

**Fig. 13:**
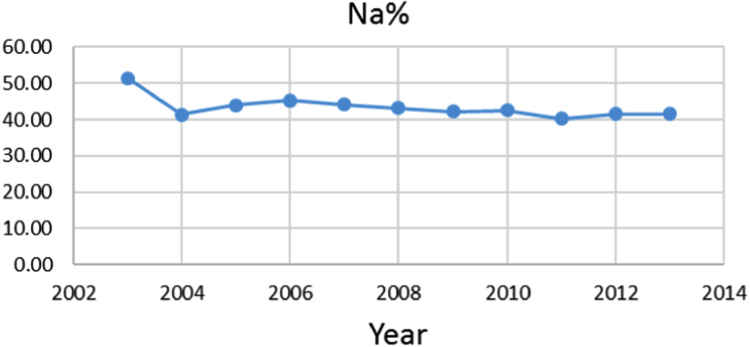
Na% index for ground water in Jolfa city (During 2003–2013).

The maximum and minimum Na% and SAR indices are 83.27, 20622 and 5.27, 0.25 respectively.

## 2 Experimental design, materials and methods

### 2.1 Study area description

Jolfa city is one of the cities of East Azerbaijan province in Iran. Jolfa city is located in East Azerbaijan province at UTM coordinates of *X*=45.17 −46.31 east longitude and *Y*=38.39 −39.2 north latitude [15]. Summer jolfa rayon is hot and dry, but winter is cold [15], [16]. Average temperature in January is between –10 and −3 °C and in July between +19 and +28 °C. Amount of annual precipitation is 200–600 mm [Fig. 1] [15], [17].

### 2.2 Data collection

The required data were collected from the results recorded in the water in the Iran Water resources management Company during the years 2003–2013. A total of 460 samples were analyzed over 11 years. Physical and chemical parameters of Jolfa city water samples were analyzed following a standard method [18], [19], [20], [21], [22], [23], [24]. Number of samples in years studied (2003–2013) presented in Table 4.

**Table 4:** Number of samples in years studied (2003–2013).

Year	Number of samples
2003	42
2004	41
2005	43
2006	42
2007	40
2008	39
2009	34
2010	80
2011	37
2012	36
2013	26
